# Incorporating Added Sugar Improves the Performance of the Health Star Rating Front-of-Pack Labelling System in Australia

**DOI:** 10.3390/nu9070701

**Published:** 2017-07-05

**Authors:** Sanne A. E. Peters, Elizabeth Dunford, Alexandra Jones, Cliona Ni Mhurchu, Michelle Crino, Fraser Taylor, Mark Woodward, Bruce Neal

**Affiliations:** 1 The George Institute for Global Health, University of Oxford, Le Gros Clark Building, South Parks Road, Oxford OX1 3QX, UK; markw@georgeinstitute.org.au; 2 Carolina Population Center, The University of North Carolina, Chapel Hill, NC 27516, USA; edunford@georgeinstitute.org.au; 3 The George Institute for Global Health, University of New South Wales, Sydney, NSW 2050, Australia; ajones@georgeinstitute.org.au (A.J.); mcrino@georgeinstitute.org.au (M.C.); ftaylor@georgeinstitute.org.au (F.T.); bneal@georgeinstitute.org.au (B.N.); 4 The Charles Perkins Centre, University of Sydney, Sydney, NSW 2006, Australia; 5 National Institute for Health Innovation, University of Auckland, Auckland 1072, New Zealand; c.nimhurchu@auckland.ac.nz; 6 School of Public Health, Faculty of Medicine, University of Sydney, Sydney, NSW 2006, Australia; 7 Department of Epidemiology, Johns Hopkins University, Baltimore, MD 21218, USA

**Keywords:** food policy, front-of-pack labelling, Health Star Rating, nutrition labels, public health

## Abstract

Background: The Health Star Rating (HSR) is an interpretive front-of-pack labelling system that rates the overall nutritional profile of packaged foods. The algorithm underpinning the HSR includes total sugar content as one of the components. This has been criticised because intrinsic sugars naturally present in dairy, fruits, and vegetables are treated the same as sugars added during food processing. We assessed whether the HSR could better discriminate between core and discretionary foods by including added sugar in the underlying algorithm. Methods: Nutrition information was extracted for 34,135 packaged foods available in The George Institute’s Australian FoodSwitch database. Added sugar levels were imputed from food composition databases. Products were classified as ‘core’ or ‘discretionary’ based on the Australian Dietary Guidelines. The ability of each of the nutrients included in the HSR algorithm, as well as added sugar, to discriminate between core and discretionary foods was estimated using the area under the curve (AUC). Results: 15,965 core and 18,350 discretionary foods were included. Of these, 8230 (52%) core foods and 15,947 (87%) discretionary foods contained added sugar. Median (Q1, Q3) HSRs were 4.0 (3.0, 4.5) for core foods and 2.0 (1.0, 3.0) for discretionary foods. Median added sugar contents (g/100 g) were 3.3 (1.5, 5.5) for core foods and 14.6 (1.8, 37.2) for discretionary foods. Of all the nutrients used in the current HSR algorithm, total sugar had the greatest individual capacity to discriminate between core and discretionary foods; AUC 0.692 (0.686; 0.697). Added sugar alone achieved an AUC of 0.777 (0.772; 0.782). A model with all nutrients in the current HSR algorithm had an AUC of 0.817 (0.812; 0.821), which increased to 0.871 (0.867; 0.874) with inclusion of added sugar. Conclusion: The HSR nutrients discriminate well between core and discretionary packaged foods. However, discrimination was improved when added sugar was also included. These data argue for inclusion of added sugar in an updated HSR algorithm and declaration of added sugar as part of mandatory nutrient declarations.

## 1. Introduction

Non-communicable diseases (NCDs) are the leading cause of death and disability globally. In 2015, 70% of all deaths were from NCDs, and of these, more than 50% were from cardiovascular disease (CVD) and diabetes [1]. Unhealthy dietary patterns and consequent diet-related metabolic risks, such as high blood pressure, dyslipidaemia, and obesity, are responsible for up to 75% of the total burden of CVD and diabetes [1].

Processed and packaged foods dominate diets in high-income countries, and their consumption in low- and middle-income countries is rapidly increasing [2,3,4]. Compared to unprocessed foods, processed foods tend to be higher in unfavourable nutrients such as added sugar, sodium, saturated fat, and trans-fats. As part of comprehensive strategies to improve diets, improving the healthiness of processed foods would, therefore, be expected to make a significant contribution to reducing the burden of diet-related NCDs [3].

Interpretive nutrition labels with simplified information on the front of packaged foods are recommended by the World Health Organization (WHO) as an evidence-based and potentially cost-effective strategy to enable consumers to make healthier choices and encourage reformulation of foods to healthier compositions by the food industry [5,6,7,8,9]. Although interpretive front-of-pack nutrition labels are proliferating worldwide, there is no international consensus on which nutrients should be included in their design to maximise their likelihood of promoting healthier diets.

The Health Star Rating (HSR) system is an Australian and New Zealand government-endorsed front-of-pack nutrition labelling system designed to make it easier for consumers to make healthier food choices [10]. The HSR system, which was implemented on a voluntary basis in 2014, assigns a rating that ranges from 0.5 (least healthy) to 5 stars (most healthy) in ten half-star increments based on the nutritional composition of a product. While the HSR system generally aligns with the Australian Dietary Guidelines [11], some inconsistencies have been identified [12,13]. A major criticism of the nutrient profiling algorithm underpinning the HSR, and a likely reason for inconsistencies with the Guidelines, is that it uses *total* sugar when calculating a product’s rating rather than added sugar. This means that intrinsic sugars, that are naturally present in dairy, fruits, and vegetables, are treated the same as sugars added during food processing. However, added sugar is widely perceived as the primary health issue and recommendations from the WHO and the Australian Dietary Guidelines focus on limiting the intake of free sugars, i.e., added sugar and natural sugar present in honeys, syrups and fruit juices but [11,14]. Added sugar is not widely listed on nutrition labels; currently, the United States is the only country that has announced mandatory added sugar labelling on the Nutrition Facts Panel for packaged foods [15].

In this study, we assessed whether using added sugar in the HSR algorithm would improve the ability of the HSR to discriminate between core and discretionary foods, and thus, would better align the HSR with the principles underpinning the Australian Dietary Guidelines.

## 2. Methods

### 2.1. Data Source

We analysed food items included in The George Institute for Global Health’s Australian FoodSwitch database [16]. This database contains nutrition label information from packaged foods available in major Australian supermarkets representing more than 90% of the Australian grocery market in 2014. For this study, we used information extracted directly from the mandatory back-of-pack nutrition information panel on energy (kJ/100 g), protein (g/100 g), saturated fat (g/100 g), total sugar (g/100 g), and sodium (mg/100 g). It is not mandatory to report details on fruit, vegetable, nut and legume (FVNL) (%), concentrated FVNL (%), and fibre (g/100 g) on the nutrition information panel. Where such details were absent, appropriate levels were estimated using information drawn from the back-of-pack ingredients list, generic food composition databases, or by analogy with similar products using methods described previously [16]. In brief, the estimation process provides a proxy value for each nutritional indicator at the category level for 728 individual food subcategories, within 18 major food categories, and this proxy value is substituted for each product in that category for which data are missing.

### 2.2. Added Sugar Values

Added sugar does not have to be reported on standard nutrition labels in Australia. Where unavailable, we extracted added sugar values from a similar food or beverage subcategory from Australian Food and Nutrient (AUSNUT) Database 2011–2013 [17]. AUSNUT is a food nutrient database containing nutrient values for 5740 generic foods and beverages with reported consumption in the 2011–2013 Australian Health Survey. Details of the procedures to allocate an amount of added sugars to each food in AUSNUT have been described elsewhere [17,18]. In short, foods were categorised in four groups based on having no sugars, only intrinsic sugars, only added sugars, or a mix of both added or free sugars and intrinsic sugars. Foods containing no sugar or only intrinsic sugars were assigned 0 g/100 g added sugar. Foods containing only added sugars were assigned an added sugar value equivalent to their total sugar value. For foods which contained a mix of added and intrinsic sugars, a recipe dataset was used to determine the added sugars values expressed in g/100 g.

### 2.3. Calculation of the Health Star Rating

The HSR was calculated in alignment with the methods described in the ‘Guide for industry to the Health Star Rating Calculator’ for all products as sold, regardless of whether a HSR was reported on the pack [19]. In short, foods were categorised into one of six product categories (i.e., non-dairy beverages; dairy beverages; oils and spreads; cheese and processed cheese; all other dairy foods; all other non-dairy foods). Baseline points were calculated based on the energy, saturated fat, total sugar, and sodium content per 100 g. Modifying points for FVNL%, concentrated FVNL%, protein, and fibre were calculated, where applicable. A HSR ‘score’ was calculated by subtracting the modifying points from baseline points. This score is then converted to a HSR based upon a defined scoring matrix for each of the six categories. The HSR ranges from 0.5 to 5.0 stars in ten half-star increments. A higher HSR reflects a healthier product. As an alternative HSR scoring system, the baseline points for sugar in the HSR algorithm were based on added sugar content (instead of total sugar content). The baseline points themselves were not changed.

### 2.4. Product Classification

Classification of products was based on the system developed by the Global Food Monitoring Group [20]. This hierarchical system is designed to monitor the nutrient composition of processed foods around the world. It classifies foods into groups (e.g., bread), categories (e.g., flat bread), and subcategories (e.g., pita bread). Food groups excluded from the current analyses were alcohol, herbs and spices, and vitamins and supplements, as these products are not required to display a nutrition label. Baby and infant foods were also excluded as they are not required to display a HSR. This left 15 major food groups used in the analysis.

### 2.5. Core and Discretionary Foods

Foods were classified as core or discretionary as per the Australian Dietary Guidelines [11,21]. Core foods are foods that form the basis of a healthy diet. In contrast, discretionary foods are energy-dense and nutrient-poor and include foods and drinks not necessary to provide the nutrients the body needs. Many of these are high in saturated fats, sugars, salt and/or alcohol.

### 2.6. Statistical Analyses

The nutritional composition of the products included were summarised within each food group, separately for core and discretionary products. Bar charts were used to assess the distribution of HSRs and the added to total sugar ratio (expressed as a percentage) across core and discretionary products. The ability of the current HSR algorithm and updated models of the HSR system to discriminate between core and discretionary foods was estimated using the area under the receiver operating characteristic curve (AUC), derived from logistic regression models. Model 1 included the HSR calculated as per current guidance [19]. In model 2, total sugar content was replaced by added sugar content when calculating the baseline points for sugar in the HSR algorithm. The continuous Net Reclassification Improvement (NRI) was calculated to estimate the proportion of discretionary products correctly assigned a higher probability and core products correctly assigned a lower probability by the updated model compared with the model for the current HSR. We also calculated the integrated discrimination improvement (IDI), which equals the difference in average predicted risks between core and discretionary products in the updated models.

Separately, we examined the capacity of the individual nutrients used in the HSR algorithm to discriminate between core and discretionary foods, without employing the HSR algorithm. We did this using a forward selection procedure based upon logistic regression. This procedure identified the nutrient per 100 g that resulted in the highest AUC at each step and built a progressively larger model until all nutrients currently included in the HSR were included (although weighted differently). For comparison, at each step, we calculated the AUC achieved by replacing the nutrient, not already in the model, that resulted in the highest increase in AUC at that step by added sugar. All analyses were conducted in R version 3.3.0 (R Foundation for Statistical Computing, Vienna, Austria).

## 3. Results

The FoodSwitch database included 15,965 core and 18,350 discretionary foods. Of these, 52% of core and 87% of discretionary foods contained added sugar. The median (Q1, Q3) HSR was 4.0 (3.0, 4.5) for core foods and 2.0 (1.0, 3.0) for discretionary foods. Median added sugar contents (g/100 g) were 3.3 (1.5, 5.5) for core foods and 14.6 (1.8, 37.2) for discretionary foods.

### 3.1. Health Star Rating within Food Groups

**Figure 1:**
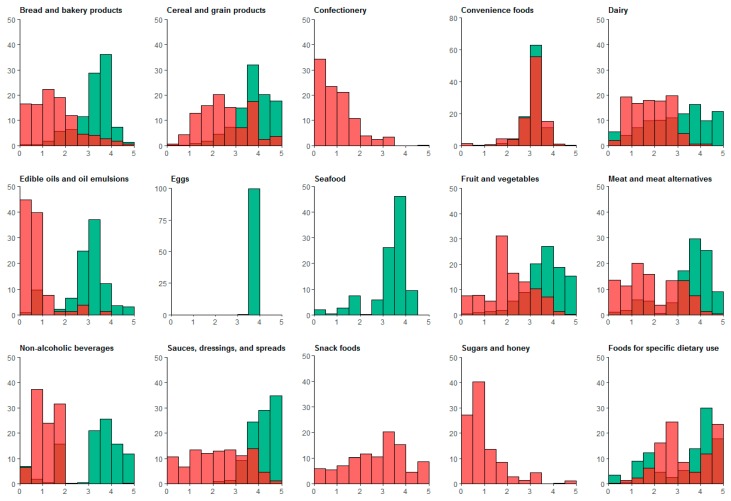
Health Star Rating for discretionary (red) and core (green) products, by food group. Discretionary products are displayed in red and core products are displayed in green. Bars are partially shaded where discretionary and core products overlap.

The median HSR was higher, indicating a healthier product, for core than for discretionary foods for all major food groups, except ‘Convenience foods’ where both core and discretionary foods had a median HSR of 3.5 (Table 1 and Table S1). However, the distribution of HSRs for core and discretionary foods overlapped for all food categories, with some core foods receiving low HSRs (≤1.5 stars) and some discretionary foods receiving high HSRs (≥4 stars) (Figure 1). Of the core foods, 17% of ‘Dairy’, 13% of ‘Foods for specific dietary use’, 10% of ‘Edible oils and oil emulsions’, 10% of ‘Beverages’, 8% of ‘Meat and meat alternatives’, and 5% of ‘Seafood’ had 1.5 stars or less. Of the discretionary foods, 40% of ‘Foods for specific dietary use’, 28% of ‘Snack foods’, 24% of ‘Cereal and grain products’, 20% of ‘Sauces, dressings and spreads’, 17% of ‘Convenience foods’, 9% of ‘Meat and meat alternatives’, 8% of ‘Fruits, vegetables, nuts and legumes’, and 5% of ‘Bread and bakery products’ had 4 or more stars.

### 3.2. Added Sugar Content within Food Groups

**Figure 2:**
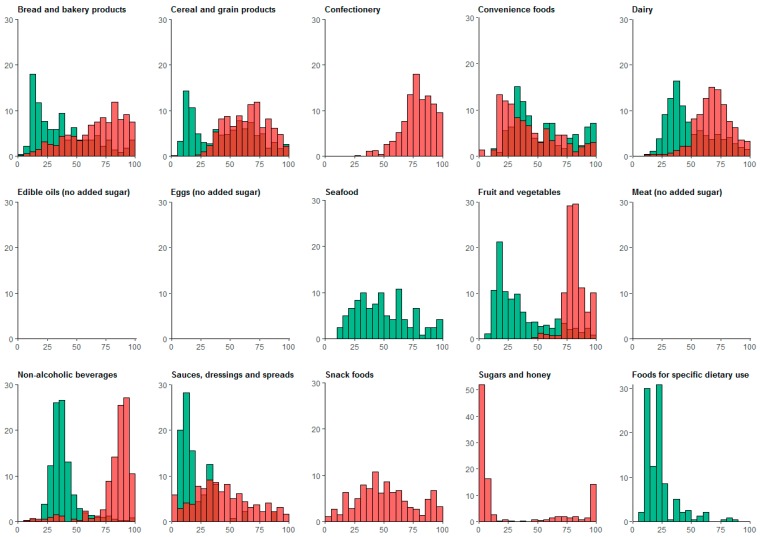
Added sugar in discretionary (red) and core (green) products, as percentage of total sugar, by food group. Discretionary products are displayed in red and core products are displayed in green. Bars are partially shaded where discretionary and core products overlap. Only products with non-zero levels of added sugar were included.

In each food group, except ‘Sauces, dressings and spreads’, a higher proportion of discretionary foods contained added sugar compared to core foods (Table 1). The median added sugar content of foods with added sugar was also higher for discretionary foods than for core foods. In discretionary foods with added sugar, median levels of added sugar were highest for ‘Foods for specific dietary use’ (57 g/100 g), ‘Fruits, vegetables, nuts and legumes’ (52 g/100 g), ‘Confectionery’ (46 g/100 g), ‘Bread and bakery products’ (17 g/100 g), ‘Dairy’ (15 g/100 g), and ‘Cereal and grain products’ (14 g/100 g). Furthermore, although the percentage of total sugar that was added was higher for discretionary than for core foods, the distributions overlapped within some food groups, particularly ‘Bread and bakery products’, ‘Cereal and grain products’, and ‘Convenience foods’ (Figure 2).

### 3.3. Discrimination between Core and Discretionary Foods

The AUC (95% confidence interval) achieved using the current HSR algorithm which incorporates total sugar was 0.825 (0.821; 0.829). The AUC increased to 0.843 (0.839; 0.847) when the baseline points for sugar in the HSR nutrient profiling algorithm were based upon added sugar (instead of total sugar). The corresponding NRI and IDI, respectively, were 0.218 (0.198; 0.237) and 0.034 (0.032; 0.036).

**Figure 3:**
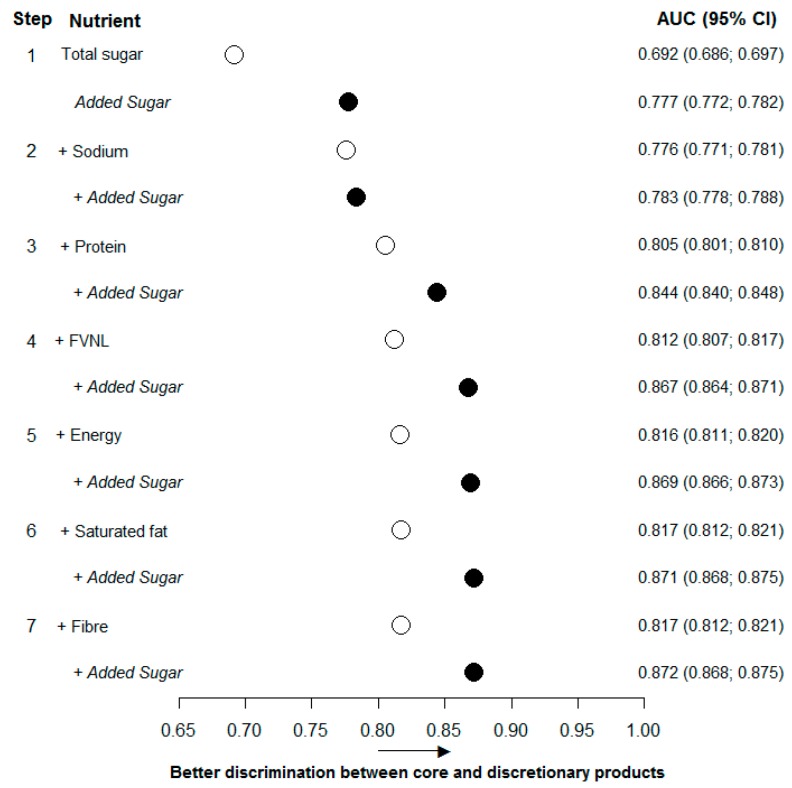
Area under the receiver operating characteristic curve to discriminate between core and discretionary foods of nutrients used in the Health Star Rating algorithm and added sugar. AUC, area under the receiver operating characteristic curve; CI, confidence interval; FVNL, fruit, vegetable, nut and legume. Circles represent the area under the receiver operating characteristic curve. Open circles are for models with nutrients used in the Health Star Rating algorithm. Filled circles are for models where the nutrient that resulted in the highest increase in area under the curve at that step was replaced by added sugar.

**Figure 4:**
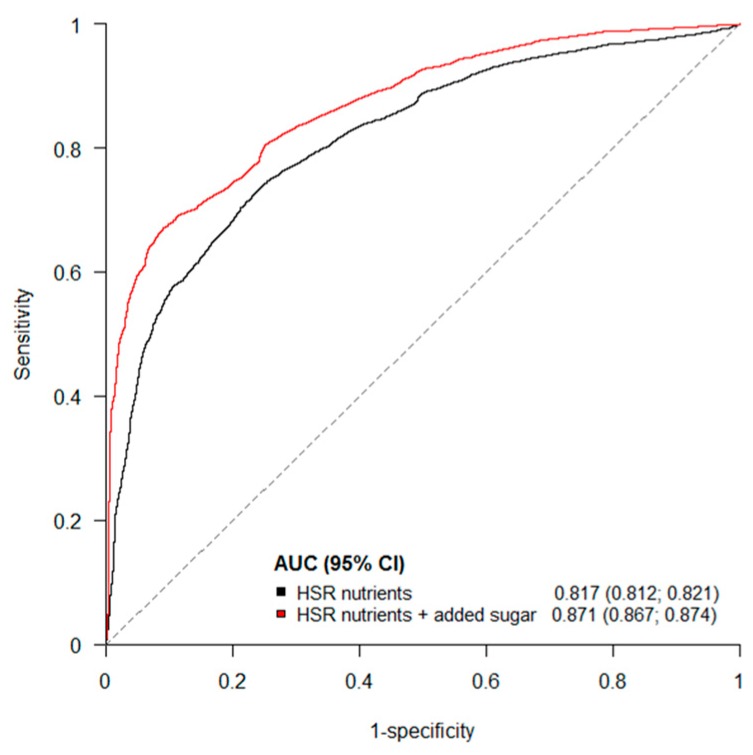
Receiver operating characteristic curve to discriminate between core and discretionary products in a model with all Health Star Rating nutrients and all Health Star Rating nutrients plus added sugar. AUC, area under the receiver operating characteristic curve; CI, confidence interval; HSR, Health Star Rating. The black curve is for a model with all HSR nutrients. The red curve is for a model with all HSR nutrients plus added sugar.

The stepwise logistic regression identified total sugar (g/100 g) as the nutrient, amongst those currently included in the HSR algorithm, with the greatest individual capacity to discriminate between core and discretionary foods: AUC 0.692 (0.686; 0.697) (Figure 3 and Table S2). In comparison, added sugar (g/100 g) alone achieved an AUC of 0.777 (0.772; 0.782). The other HSR nutrients that increased the AUC most when included in the model alongside total sugar were, in order of effect, sodium, protein, FVNL%, energy, saturated fat, and fibre. At every step, the inclusion of added sugar resulted in a substantially greater AUC (Figure 3). The full logistic regression model, including all nutrients currently included in the HSR algorithm, achieved an AUC of 0.817 (0.812; 0.821), which increased to 0.871 (0.867; 0.874) when added sugar was added to the model (Figure 4 and Table S2).

## 4. Discussion

This analysis of over 34,000 packaged foods and drinks available in Australian supermarkets indicates that the HSR front-of-pack labelling system is broadly aligned with Australian Dietary Guidelines. However, switching added sugar for total sugar in the HSR algorithm leads to better discrimination between core and discretionary foods.

The HSR system is intended to provide consumers with a quick summary of the nutritional quality of packaged foods and drinks that is easy to understand and is consistent with dietary guidelines [10]. In line with this study’s findings, previous analyses have shown that the HSR system assigns higher ratings to foods that form the basis of a healthy diet (i.e., core foods) and lower ratings to foods that can be included occasionally in small amounts, but are not a necessary part of the diet (i.e., discretionary foods) [12,13,22]. A two-year review of the system suggested that the HSR has had significant uptake and was displayed on over 5500 products in Australia and over 800 products in New Zealand in 2016 [23]. Consumer awareness and use of HSR are also increasing, and there is some evidence that the HSR encourages manufacturers to reformulate their products to obtain a higher star rating [23,24]. Recent research by an Australian consumer organisation indicated that many consumers find the HSR useful and want to see it rolled out across a wider range of products [25]. This notwithstanding, there remains significant consumer concern about apparent anomalies in the HSR system, particularly those that allow products with high levels of added sugar, sodium, or saturated fat, and few positive nutrients, to display relatively high HSRs. Even if anomalies exist for only a small number of products, they could have a major impact on public confidence and trust in the HSR system, ultimately undermining its utility and effectiveness overall. For this reason, the HSR Advisory Committee has established a Technical Advisory Group (TAG) to assist in review of the HSR algorithm as part of a formal five-year review [23]. The findings of the present study are directly relevant to the TAG’s mandate to consider whether the star ratings being produced by the HSR algorithm align with the Australian Dietary Guidelines. Our results suggest that including added sugar in the HSR algorithm would enhance this alignment.

Incorporating added sugar into the HSR algorithm would also reflect robust and growing evidence of the harms caused by high intake of added sugar. Dietary guidelines and the WHO recommend limiting dietary intake of added sugars because of the substantial health risks of overweight and obesity as well as poor dental health [11,14]. However, food manufacturers in Australia are currently only required to list total sugar on nutrition labels and specification of added sugar is not required [26]. As such, consumers must read complex product ingredient lists to discern how much of the total sugar is intrinsic versus added. This can be challenging, even for nutritionists, given the wide variety of terms used for added sugar. A recent survey in Australia and New Zealand reported a strong consumer desire to have information on added sugar available on nutrition labels and ingredient lists [25]. Following an independent review of food labelling, Food Standards Australia New Zealand (FSANZ), in consultation with the Food Regulation Standing Committee (FRSC), are currently preparing a programme of work to further investigate labelling approaches for providing information on sugars [27]. Similar evaluations of labelling requirements are ongoing in Europe and North America. In the United States, the Food and Drug Administration (FDA) recently finalised the new ‘Nutrition Facts Panel’ for packaged foods, which, from 2018, requires food manufacturers to include total and added sugars on the label [15]. Arguably, the most significant impact of any labelling initiative will be delivered through its capacity to generate a food-systems response to improve the nutrient content of the food supply as a whole. Given our findings, and widespread consumer demand for clearer information on added sugars, inclusion of added sugars in both the nutrient declaration and HSR algorithm may incentivise the food industry to undertake food reformulation, improving the healthiness of the food supply for all [28].

Some limitations of this study need to be mentioned. First, information on added sugar, FVNL content, and fibre are not currently mandatory on standard back-of-pack nutrition information panel in Australia and thus, missing values were estimated from ingredients lists, food composition databases, and other sources. Since a considerable proportion of products contain a mixture of added and intrinsic sugars, this may have led to discrepancies between the HSR calculated using the actual and imputed values (Table S3). If the misclassification is not differential, this will likely have resulted in underestimation of the ability of added sugar to discriminate between core and discretionary products. Second, we directly applied the baseline points for total sugar in the current HSR nutrient profiling algorithm to added sugar, without recalibrating the scoring system. As illustrated by our analyses on the individual HSR nutrients from first principles, which assigned values independent of the nutrient profiling algorithm, this has likely underestimated the discriminatory value of added sugar in relation to what foods are core, rather than discretionary. Third, our analyses were restricted to packaged foods available in supermarkets in Australia, for which the HSR system is currently designed. Future studies should assess the useability and transferability of the HSR system to fresh produce, food service and restaurant foods, and foods available in other countries.

## 5. Conclusions

In conclusion, the current HSR nutrients discriminate well between core and discretionary packaged foods. However, discrimination was improved when added sugar was also included. These data argue for the inclusion of added sugar in an updated HSR algorithm and declaration of added sugar as part of mandatory nutrient declarations.

## Figures and Tables

**Table 1:** Health Star Rating and added sugar content across food groups.

Food Group	*N* (% Discretionary)	HSR *		Total Sugar, g/100 g *		Has Added Sugar, %		Added Sugar, g/100 g *^,†^	
		Core	Discretionary	Core	Discretionary	Core	Discretionary	Core	Discretionary
Bread and bakery products	4021 (64)	3.5 (3.0; 4.0)	1.5 (1.0; 2.5)	3 (1; 4)	25 (5; 35)	43	99	1 (1; 2)	17 (3; 31)
Cereal and grain products	3254 (22)	4.0 (3.5; 4.5)	2.5 (2.0; 3.5)	3 (1; 14)	25 (18; 31)	59	100	3 (1; 11)	14 (12; 23)
Confectionery	3096 (100)	NA	1.0 (0.5; 1.5)	NA	53 (43; 60)	NA	100	NA	46 (43; 49)
Convenience foods	1602 (54)	3.5 (3.5; 3.5)	3.5 (3.0; 3.5)	3 (2; 5)	3 (2; 4)	86	84	1 (0; 3)	1 (0; 1)
Dairy	4566 (28)	3.5 (2.0; 4.0)	2.0 (1.5; 3.0)	5 (1; 10)	21 (16; 25)	56	87	4 (0; 5)	15 (14; 19)
Edible oils and oil emulsions	689 (23)	3.5 (3.0; 3.5)	1.0 (0.5; 1.0)	1 (1; 1)	1 (1; 1)	0	0	NA	NA
Eggs	209 (0)	4.0 (4.0; 4.0)	NA	0 (0; 0)	NA	0	NA	NA	NA
Seafood	1309 (0)	4.0 (3.5; 4.0)	NA	1 (1; 2)	NA	39	NA	2 (2; 2)	NA
Fruit, vegetables, nuts, and legumes	4227 (26)	4.0 (3.5; 4.5)	2.0 (2.0; 3.0)	6 (3; 15)	26 (3; 59)	39	40	8 (5; 11)	52 (49; 52)
Meat and meat alternatives	1955 (68)	4.0 (3.5; 4.5)	2.0 (1.5; 3.0)	1 (1; 2)	1 (1; 1)	48	64	1 (1; 8)	1 (0; 1)
Non-alcoholic beverages	3101 (45)	4.0 (2.0; 4.5)	1.5 (1.0; 2.0)	9 (7; 11)	10 (6; 11)	68	71	3 (3; 4)	10 (9; 10)
Sauces, dressings and spreads	3588 (95)	4.5 (4.0; 5.0)	2.5 (1.5; 3.5)	7 (4; 11)	7 (3; 21)	100	93	2 (1; 6)	5 (1; 16)
Snack foods	1328 (100)	NA	3.0 (2.0; 4.0)	NA	4 (2; 7)	NA	88	NA	2 (1; 5)
Foods for specific dietary use ^‡^	598 (54)	4.0 (2.0; 4.5)	3.0 (2.5; 4.5)	8 (7; 36)	6 (3; 18)	100	100	5 (2; 5)	57 (57; 57)
Sugars, honey and related products	772 (100)	NA	1.0 (0.5; 1.5)	NA	82 (60; 87)	100	100	NA	5 (1; 36)

HSR, Health Star Rating; NA, not applicable. * Values are presented as median (25th percentile; 75th percentile). ^†^ calculated in products with non-zero levels as derived from the Australian Food and Nutrient Database (AUSNUT). **^‡^** This category includes diet drink mixes, meal replacements, breakfast beverages, sports gels, protein and diet bars, and breakfast beverages.

## Supplementary Materials

The following are available online at www.mdpi.com/2072-6643/9/7/701/s1, Table S1: Nutritional composition of included foods, by food group; Table S2: Area under the receiver operating characteristic curve to discriminate between core and discretionary foods of nutrients used in the Health Star Rating algorithm and added sugar; Table S3: Percentage of foods with no sugars, only intrinsic sugars, only added sugars, or a mix of both added or free sugars and intrinsic sugars, by food group.
