# Supplementary material for: Incorporating Added Sugar Improves the Performance of the Health Star Rating Front-of-Pack Labelling System in Australia
Source: Nutrients. 2017 Jul 5;9(7):701. doi: 10.3390/nu9070701 (PMC5537816; doi:10.3390/nu9070701)
Supplement: Supplementary file 1 [file nutrients-09-00701-s001.zip › nutrients-203332-supplementary.pdf]

Supplementary Materials: Incorporating Added Sugar Improves the Performance of the Health Star Rating Front-of-Pack Labelling System in Australia

Sanne A. E. Peters, Elizabeth Dunford, Alexandra Jones, Cliona Ni Mhurchu, Michelle Crino, Fraser Taylor, Mark Woodward and Bruce Neal

Table S1. Nutritional composition of included foods, by food group.

	n	Energy	Saturated fat		Sugars		Sodium		FVNL		Fibre		Protein		HSR	Added sugar*	
		kJ/100g	%	g/100g	%	g/100g	%	g/100g	%	%/100g	%	g/100g	%	g/100g		%	g/100g
**Bread and bakery products**																	
All	4021	1630 (1170; 1900)	99	3 (1; 9)	99	7 (2; 30)	100	372 (230; 520)	3	22 (22; 22)	98	4 (3; 5)	100	7 (5; 9)	2.0 (1.5; 3.5)	79	16 (2; 30)
Core	1449	1110 (1010; 1330)	100	1 (0; 1)	99	3 (1; 4)	100	400 (360; 542)	0	NA	100	4 (4; 5)	100	9 (7; 10)	3.5 (3.0; 4.0)	43	1 (1; 2)
Discretionary	2572	1800 (1555; 2016)	99	7 (3; 12)	99	25 (5; 35)	100	300 (185; 490)	4	22 (22; 22)	97	3 (2; 5)	100	6 (5; 8)	1.5 (1.0; 2.5)	99	17 (3; 31)
**Cereal and grain products**																	
All	3254	1550 (1470; 1720)	96	1 (1; 3)	95	8 (2; 21)	98	39 (10; 224)	31	15 (13; 31)	97	7 (4; 9)	100	10 (7; 12)	4.0 (3.0; 4.5)	68	11 (2; 14)
Core	2526	1520 (1445; 1620)	95	1 (0; 2)	94	3 (1; 14)	98	30 (7; 235)	27	31 (15; 31)	97	7 (4; 10)	100	11 (8; 13)	4.0 (3.5; 4.5)	59	3 (1; 11)
Discretionary	728	1740 (1610; 1900)	99	4 (2; 7)	100	25 (18; 31)	100	102 (32; 200)	44	13 (13; 13)	100	6 (5; 8)	100	8 (6; 10)	2.5 (2.0; 3.5)	100	14 (12; 23)
**Confectionery**																	
All (all discretionary)	3096	1980 (1530; 2230)	91	14 (2; 19)	97	53 (43; 60)	97	60 (25; 95)	9	38 (25; 38)	56	6 (4; 11)	92	5 (3; 7)	1.0 (0.5; 1.5)	100	46 (43; 49)
**Convenience foods**																	
All	1602	485 (314; 731)	97	1 (1; 3)	99	3 (2; 4)	100	290 (240; 391)	79	42 (25; 45)	72	1 (1; 2)	100	5 (3; 8)	3.5 (3.0; 3.5)	85	1 (0; 1)
Core	729	369 (207; 690)	95	1 (0; 2)	99	3 (2; 5)	100	276 (235; 351)	88	45 (29; 48)	58	1 (1; 1)	100	3 (1; 6)	3.5 (3.5; 3.5)	86	1 (0; 3)
Discretionary	873	541 (406; 758)	99	2 (1; 3)	99	3 (2; 4)	100	305 (244; 430)	72	28 (13; 44)	84	1 (1; 2)	100	6 (5; 10)	3.5 (3.0; 3.5)	84	1 (0; 1)
**Dairy**																	
All	4566	628 (352; 1280)	98	4 (2; 15)	98	8 (3; 16)	100	60 (44; 280)	11	7 (7; 7)	2	1 (1; 1)	100	4 (3; 9)	3.0 (2.0; 4.0)	65	6 (4; 14)
Core	3283	494 (300; 1270)	99	3 (1; 16)	97	5 (1; 10)	100	63 (45; 570)	15	7 (7; 7)	0	NA	100	5 (3; 17)	3.5 (2.0; 4.0)	56	4 (0; 5)
Discretionary	1283	903 (640; 1290)	95	8 (3; 12)	100	21 (16; 25)	100	52 (35; 77)	0	NA	6	1 (1; 1)	99	3 (2; 4)	2.0 (1.5; 3.0)	87	15 (14; 19)
**Edible oils and oil emulsions**																	
All	689	3378 (2610; 3436)	100	15 (13; 33)	47	1 (1; 1)	59	340 (5; 380)	0	NA	0	NA	47	1 (1; 1)	3.0 (1.0; 3.5)	0	NA
Core	531	3390 (2420; 3446)	100	14 (13; 16)	34	1 (1; 1)	50	340 (5; 360)	0	NA	0	NA	34	1 (1; 1)	3.5 (3.0; 3.5)	0	NA
Discretionary	158	3025 (3000; 3080)	100	53 (35; 55)	91	1 (1; 1)	91	375 (19; 640)	0	NA	0	NA	92	1 (1; 1)	1.0 (0.5; 1.0)	0	NA
**Eggs**																	
All (all core)	209	559 (559; 559)	99	3 (3; 3)	100	0 (0; 0)	100	136 (136; 136)	0	NA	0	NA	100	12 (12; 12)	4.0 (4.0; 4.0)	0	NA
**Seafood**																	
All (all core)	1309	687 (482; 855)	98	1 (1; 2)	81	1 (1; 2)	100	393 (290; 520)	0	NA	0	NA	100	18 (13; 22)	4.0 (3.5; 4.0)	39	2 (2; 2)
**Fruit, vegetables, nuts and legumes**																	
All	4227	884 (266; 1640)	84	1 (0; 4)	97	7 (3; 30)	97	31 (6; 300)	89	78 (61; 85)	94	5 (2; 7)	98	3 (1; 9)	3.5 (3.0; 4.5)	39	11 (5; 18)
Core	3137	597 (243; 2171)	87	1 (0; 5)	98	6 (3; 15)	98	21 (5; 230)	96	85 (67; 100)	95	6 (2; 7)	99	3 (1; 12)	4.0 (3.5; 4.5)	39	8 (5; 11)
Discretionary	1090	992 (553; 1150)	75	1 (0; 2)	95	26 (3; 59)	95	348 (13; 1600)	68	56 (44; 78)	89	2 (1; 9)	96	1 (1; 3)	2.0 (2.0; 3.0)	40	52 (49; 52)
**Meat and meat alternatives**																	
All	1955	868 (601; 1060)	100	4 (2; 7)	97	1 (1; 2)	100	569 (364; 970)	8	81 (81; 81)	33	2 (2; 3)	100	16 (12; 20)	3.0 (1.5; 4.0)	59	1 (0; 1)
Core	616	648 (493; 858)	100	2 (1; 4)	98	1 (1; 2)	100	330 (129; 498)	25	81 (81; 81)	44	2 (1; 3)	100	17 (13; 20)	4.0 (3.5; 4.5)	48	1 (1; 8)
Discretionary	1339	954 (700; 1120)	100	6 (3; 8)	97	1 (1; 1)	100	759 (474; 1180)	0	NA	29	2 (2; 2)	100	16 (12; 20)	2.0 (1.5; 3.0)	64	1 (0; 1)
**Beverages**																	
All	3101	177 (105; 200)	34	1 (0; 1)	88	9 (7; 11)	94	8 (5; 15)	41	88 (87; 96)	1	28 (28; 28)	63	1 (0; 1)	2.0 (1.5; 4.0)	69	4 (3; 10)

Core	1704	184 (156; 210)	45	1 (0; 1)	92	9 (7; 11)	93	6 (4; 10)	74	88 (87; 96)	2	28 (28; 28)	86	1 (0; 1)	4.0 (2.0; 4.5)	68	3 (3; 4)
Discretionary	1397	148 (64; 192)	20	1 (0; 1)	83	10 (6; 11)	96	11 (6; 18)	0	NA	0	NA	36	1 (0; 1)	1.5 (1.0; 2.0)	71	10 (9; 10)
**Sauces, dressings and spreads**																	
All	3588	624 (330; 1120)	86	1 (0; 4)	98	7 (3; 20)	99	525 (330; 990)	66	98 (25; 100)	64	2 (1; 4)	98	2 (1; 4)	2.5 (1.5; 3.5)	94	5 (1; 16)
Core	196	2470 (407; 2626)	97	7 (1; 9)	98	7 (4; 11)	99	68 (11; 431)	100	100 (100; 100)	100	6 (4; 10)	99	21 (4; 24)	4.5 (4.0; 5.0)	100	2 (1; 6)
Discretionary	3392	604 (328; 1060)	85	1 (0; 3)	98	7 (3; 21)	99	545 (350; 1055)	64	40 (25; 100)	62	2 (1; 4)	98	2 (1; 4)	2.5 (1.5; 3.5)	93	5 (1; 16)
**Snack foods**																	
All (all discretionary)	1328	2040 (1824; 2160)	99	3 (2; 10)	97	4 (2; 7)	100	560 (400; 785)	82	100 (100; 100)	91	3 (3; 6)	100	7 (6; 9)	3.0 (2.0; 4.0)	88	2 (1; 5)
**Foods for specific dietary use**																	
All	598	1490 (346; 1610)	97	3 (1; 6)	100	8 (4; 22)	100	180 (65; 290)	0	NA	71	11 (6; 11)	98	25 (6; 32)	3.8 (2.5; 4.5)	100	57 (5; 57)
Core	276	342 (290; 1584)	99	1 (0; 2)	100	8 (7; 36)	100	81 (65; 327)	0	NA	43	6 (6; 6)	100	7 (4; 28)	4.0 (2.0; 4.5)	100	5 (2; 5)
Discretionary	322	1544 (1436; 1662)	95	6 (5; 8)	100	6 (3; 18)	100	190 (130; 267)	0	NA	95	11 (11; 11)	97	29 (22; 33)	3.0 (2.5; 4.5)	100	57 (57; 57)
**Sugars, honey and related products**																	
All (all discretionary)	772	1416 (1340; 1670)	40	1 (0; 3)	96	82 (60; 87)	90	15 (6; 35)	0	NA	0	NA	74	0 (0; 1)	1.0 (0.5; 1.5)	100	5 (1; 36)

HSR, Health Star Rating. Values are presented as median (25th percentile; 75th percentile). % indicates percentage of products that displayed a value. *calculated in products with non-zero levels, as derived from AUSNUT.

Table S2. Area under the receiver operating characteristic curve to discriminate between core and discretionary foods of nutrients used in the Health Star Rating algorithm and added sugar.

	**Nutrient added from previous step**							
	**Step 1**	**Step 2**	**Step 3**	**Step 4**	**Step 5**	**Step 6**	**Step 7**	**Step 8**
	NA	*Total sugar*	*+ Sodium*	*+ Protein*	*+ FVNL*	*+ Energy*	*+ Saturated fat*	*+ Fibre*
**Nutrient to be added (if not added already)**								
Energy	0.614 (0.608; 0.620)	0.693 (0.687; 0.698)	0.775 (0.771; 0.780)	0.809 (0.804; 0.813)	**0.816 (0.811; 0.820)**	In model	In model	In model
Saturated fat	0.587 (0.580; 0.593)	0.699 (0.693; 0.704)	0.779 (0.774; 0.784)	0.809 (0.805; 0.814)	0.815 (0.811; 0.820)	**0.817 (0.812; 0.821)**	In model	In model
Total sugar	**0.692 (0.686; 0.697)**	In model	In model	In model	In model	In model	In model	In model
Sodium	0.602 (0.596; 0.608)	**0.776 (0.771; 0.781)**	In model	In model	In model	In model	In model	In model
FVNL	0.569 (0.564; 0.574)	0.710 (0.705; 0.716)	0.773 (0.768; 0.778)	**0.812 (0.807; 0.817)**	In model	In model	In model	In model
Fibre	0.499 (0.493; 0.505)	0.694 (0.688; 0.699)	0.775 (0.770; 0.780)	0.805 (0.800; 0.810)	0.812 (0.808; 0.817)	0.816 (0.811; 0.820)	**0.817 (0.812; 0.821)**	In model
Protein	0.620 (0.614; 0.626)	0.716 (0.711; 0.722)	**0.805 (0.801; 0.810)**	In model	In model	In model	In model	In model
Added sugar	*0.777 (0.772; 0.782)*	*0.783 (0.778; 0.788)*	*0.844 (0.840; 0.848)*	*0.867 (0.864; 0.871)*	*0.869 (0.866; 0.873)*	*0.871 (0.868; 0.875)*	*0.872 (0.868; 0.875)*	***0.871 (0.867; 0.874)***

FVNL, Fruit, vegetable, nut and legume; HSR, Health Star Rating. Values represent the area under the receiver operating characteristic curve (95% confidence interval). Printed in bold are the results for the nutrient that resulted in the highest AUC at each step. Models including added sugar are printed in italics.

Table S3. Percentage of foods with no sugars, only intrinsic sugars, only added sugars, or a mix of both added or free sugars and intrinsic sugars, by food group.

**Bread and bakery products**	**N**	**No sugars, %**	**Only intrinsic sugars, %**	**Only added sugars, %**	**Mix of added and intrinsic sugars, %**
Core	1449	0	57	1	43
Discretionary	2572	0	1	1	98
**Cereal and grain products**					
Core	2526	5	36	1	58
Discretionary	728	0	0	0	100
**Confectionery**					
Discretionary	3096	0	0	3	97
**Convenience foods**					
Core	729	0	14	1	85
Discretionary	873	0	16	1	83
**Dairy**					
Core	3283	2	42	0	55
Discretionary	1283	0	13	0	87
**Edible oils and oil emulsions**					
Core	531	66	34	0	0
Discretionary	158	9	91	0	0
**Eggs**					
Core	209	0	100	0	0
**Seafood**					
Core	1309	11	50	8	31
**Fruit, vegetables, nuts and legumes**					
Core	3137	1	60	1	38
Discretionary	1090	5	55	0	40
**Meat and meat alternatives**					
Core	616	2	50	0	48
Discretionary	1339	1	34	1	63
**Beverages**					
Core	1704	8	25	1	67
Discretionary	1397	4	25	13	58
**Sauces, dressings, and spreads**					
Core	196	0	0	2	98
Discretionary	3392	0	6	2	92
**Snack foods**					
Discretionary	1328	1	11	2	86
**Foods for specific dietary use**					
Core	276	0	0	0	100
Discretionary	322	0	0	0	100
**Sugars, honey and related products**					
Discretionary	772	0	0	4	96
